# Author Correction: Distinct molecular and immune hallmarks of inflammatory arthritis induced by immune checkpoint inhibitors for cancer therapy

**DOI:** 10.1038/s41467-024-49733-9

**Published:** 2024-07-04

**Authors:** Sang T. Kim, Yanshuo Chu, Mercy Misoi, Maria E. Suarez-Almazor, Jean H. Tayar, Huifang Lu, Maryam Buni, Jordan Kramer, Emma Rodriguez, Zulekha Hussain, Sattva S. Neelapu, Jennifer Wang, Amishi Y. Shah, Nizar M. Tannir, Matthew T. Campbell, Don L. Gibbons, Tina Cascone, Charles Lu, George R. Blumenschein, Mehmet Altan, Bora Lim, Vincente Valero, Monica E. Loghin, Janet Tu, Shannon N. Westin, Aung Naing, Guillermo Garcia-Manero, Noha Abdel-Wahab, Hussein A. Tawbi, Patrick Hwu, Isabella C. Glitza Oliva, Michael A. Davies, Sapna P. Patel, Jun Zou, Andrew Futreal, Adi Diab, Linghua Wang, Roza Nurieva

**Affiliations:** 1 Section of Rheumatology and Clinical Immunology, Department of General Internal Medicine, The University of Texas MD Anderson Cancer Center, Houston, TX 77030 USA; 2 Department of Genomic Medicine, The University of Texas MD Anderson Cancer Center, Houston, TX 77030 USA; 3 Department of General Internal Medicine, Baylor College of Medicine, Houston, TX 77030 USA; 4 Department of Immunology, The University of Texas MD Anderson Cancer Center, Houston, TX 77030 USA; 5 Department of Biology, Georgetown University, Washington, DC 20057 USA; 6 Department of Infectious Disease, Infection Control and Employee Health, The University of Texas MD Anderson Cancer Center, Houston, TX 77030 USA; 7 Department of Lymphoma and Myeloma, The University of Texas MD Anderson Cancer Center, Houston, TX 77030 USA; 8 Department of Genitourinary Medical Oncology, The University of Texas MD Anderson Cancer Center, Houston, TX 77030 USA; 9 Department of Thoracic Head and Neck Medical Oncology, The University of Texas MD Anderson Cancer Center, Houston, TX 77030 USA; 10 Department of Breast Medical Oncology, The University of Texas MD Anderson Cancer Center, Houston, TX 77030 USA; 11 Department of Neuro-Oncology, The University of Texas MD Anderson Cancer Center, Houston, TX 77030 USA; 12 Department of General Oncology, The University of Texas MD Anderson Cancer Center, Houston, TX 77030 USA; 13 Department of Gynecologic Oncology and Reproductive Medicine, The University of Texas MD Anderson Cancer Center, Houston, TX 77030 USA; 14 Department of Investigational Cancer Therapeutics, The University of Texas MD Anderson Cancer Center, Houston, TX 77030 USA; 15 Department of Leukemia, The University of Texas MD Anderson Cancer Center, Houston, TX 77030 USA; 16 Department of Melanoma Medical Oncology, The University of Texas MD Anderson Cancer Center, Houston, TX 77030 USA; 17 Department of Rheumatology and Rehabilitation, Assiut University Hospitals, Faculty of Medicine, Assiut University, El Fateh, Egypt; 18 Department of Laboratory Medicine, The University of Texas MD Anderson Cancer Center, Houston, TX 77030 USA; 19 The University of Texas MD Anderson Cancer Center UTHealth Graduate School of Biomedical Sciences (GSBS), Houston, TX 77030 USA; 20 Present Address: H. Lee Moffitt Cancer Center and Research Institute, Tampa, FL 33612 USA

**Keywords:** Tumour immunology, Autoimmunity, Tumour immunology

Correction to: *Nature Communications* 10.1038/s41467-022-29539-3, published online 12 April 2022

In this article the funding information for Jordan Kramer was omitted and should have read, ‘J.K. is supported by the CPRIT Research Training Award CPRIT Training Program (RP210028)’. The original article has been corrected.

