# Supplementary material for: Distinct molecular and immune hallmarks of inflammatory arthritis induced by immune checkpoint inhibitors for cancer therapy
Source: Nat Commun. 2022 Apr 12;13:1970. doi: 10.1038/s41467-022-29539-3 (PMC9005525; doi:10.1038/s41467-022-29539-3)
Supplement: Supplementary file 1 — Supplementary information [file 41467_2022_29539_MOESM1_ESM.pdf]

Supplementary Figure 1

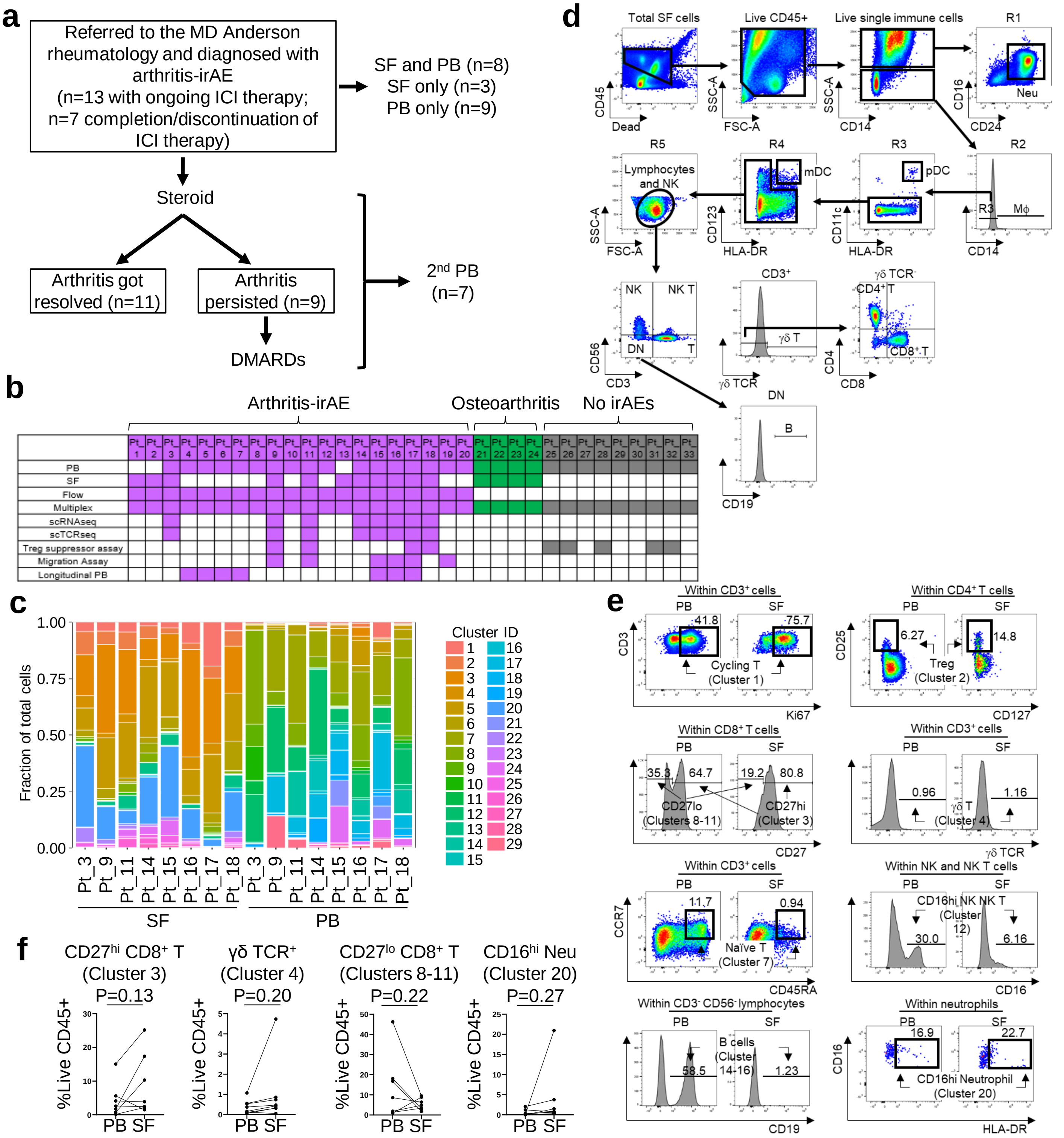

Supplementary Figure 1. Molecular and immunologic analysis of patients with arthritis as an immune-related adverse event (arthritis-irAE). (**a-b**) Collection of peripheral blood (PB) and/or synovial fluid (SF) samples. PB and/or SF samples were obtained from 20 patients with arthritis-irAE at the time of the active arthritis. We also collected the 2nd PB samples from 7 patients during arthritis-irAE treatment. The samples were analyzed with flow cytometry, single cell RNA sequencing (scRNAseq), single cell T cell receptor sequencing (scTCRseq), multiplex, in vitro regulatory T cell (Treg) suppressor assay, and/or migration assay. As a negative control, SF and PB samples were collected from 4 immune checkpoint inhibitor–naïve patients with osteoarthritis, and PB samples were collected from 9 patients who tolerated immune checkpoint inhibitors well for at least for 12 weeks. (**c**) Fraction of 29 cell clusters in Fig. 1b across individual patients. (**d**) Gating strategy for flow cytometry analysis. Live immune cells were detected by gating CD45 and live-dead. One of the most representative plots from an SF sample is shown. FSC-A, forward scatter area; SSC-A, side scatter area; Mϕ, macrophages; neu, neutrophils; mDC, myeloid dendritic cells; pDC, plasmacytoid dendritic cells; NK, natural killer cells; DN, double negative, B, B cells; T, T cells. Corresponds to Figure 1. (**e-f**) Flow cytometry plots to identify cell populations matching with clusters in Fig. 1 (e) and quantitative analysis (f). n=7 (CD27hi CD8+ T; CD27lo CD8+ T; CD16hi Neu) or n=8 (γδ TCR+) PB and matching SF samples. Two-sided paired t-test. PB, peripheral blood; SF, synovial fluid. Source data are provided as a Source Data file.

Supplementary Figure 2

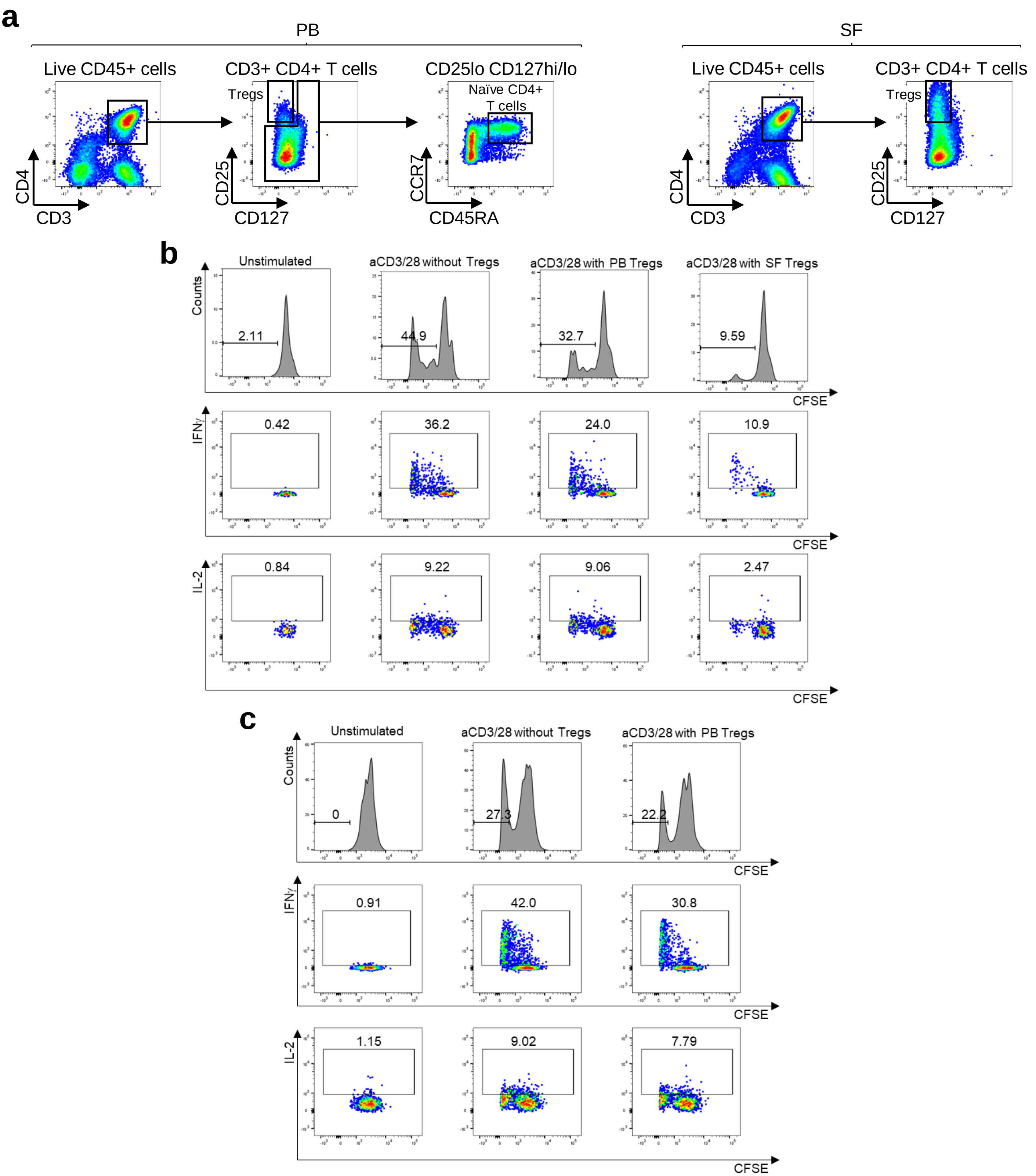

Supplementary Figure 2. Representative flow cytometry plots of in vitro regulatory T cell (Treg) suppressor assay. (**a**) Flow cytometry plots showing sorting strategies. PB, peripheral blood; SF, synovial fluid. Corresponds to Figure 3. (**b**) 10,000 CFSE-stained PB naïve CD4+ T cells were stimulated with anti-CD3/28 in the absence of autologous Tregs or in the presence of 20,000 autologous Tregs from PB or SF for 4 days. The percentage of CFSE-negative, interferon gamma (IFNγ)-producing, and interleukin-2 (IL-2)-producing populations within naïve CD4+ T cells is shown. (**c**) Parallel experiments were performed using PB samples from age-, sex-, and tumor-matched patients who did not develop immune-related adverse events at least 12 weeks after initiating immune checkpoint inhibitor therapy.

Supplementary Figure 3

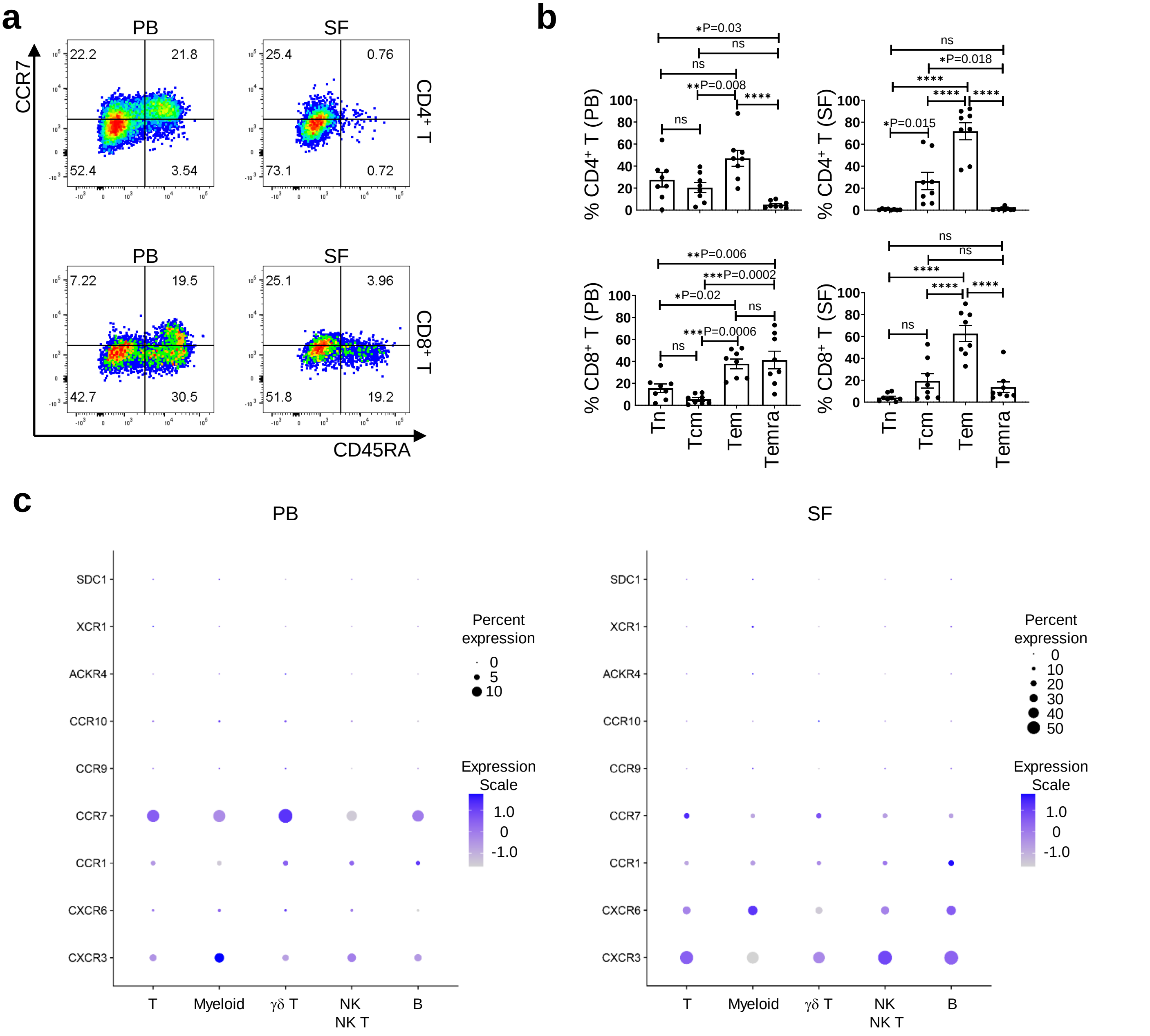

Supplementary Figure 3. Analysis of T cell subsets. (**a-b**) Representative flow cytometry plots of CD45RA and CCR7 staining of CD4+ and CD8+ T cells (**a**) and quantification analyses (**b**). One-way analysis of variance. *P<0.05, **P<0.01, ***P<0.001, ****P<0.0001. Bars indicate the mean and SEM. PB, peripheral blood; SF, synovial fluid; Tn, naïve T cells; Tcm, central memory T cells, Tem, effector memory T cells; Temra, terminally differentiated effector memory T cells. n=8 PB samples and 8 SF samples. (**c**) Bubble plots to show chemokine receptors highly expressed on immune cell subsets. PB, peripheral blood; SF, synovial fluid; NK, natural killer cells; NK T, natural killer T cells. Source data are provided as a Source Data file.

Supplementary Figure 4

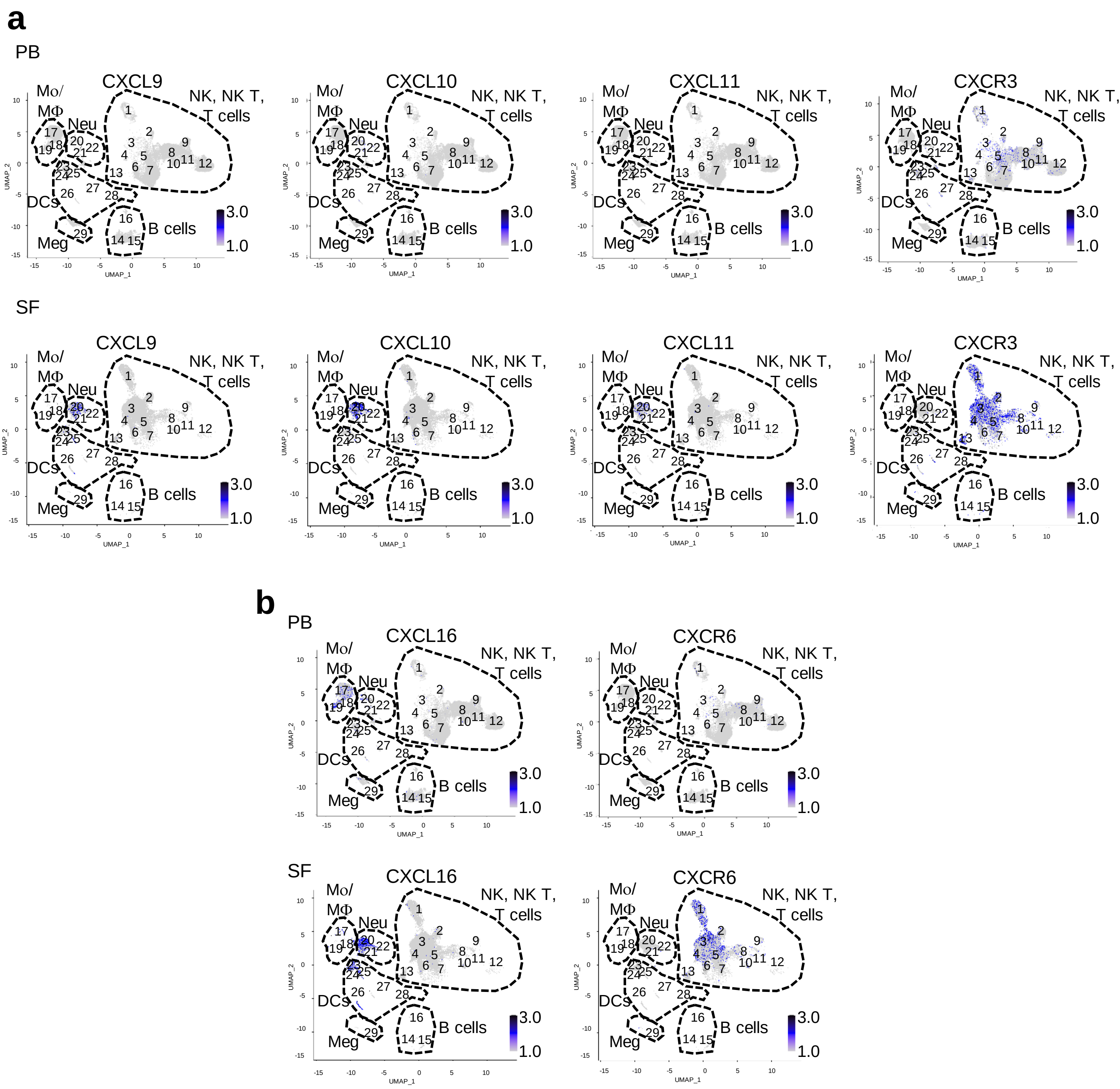

Supplementary Figure 4. Expression of CXCL9/10/11-CXCR3 and CXCL16-CXCR6 on live cells. (**a-b**) Feature plots showing expression of CXCL9/10/11-CXCR3 (**a**) and CXCL16-CXCR6 (**b**) on live cells. Cell clusters and their annotations are identical to those in Fig. 1. Created by authors using R 3.6.0. PB, peripheral blood; SF, synovial fluid.

Supplementary Figure 5

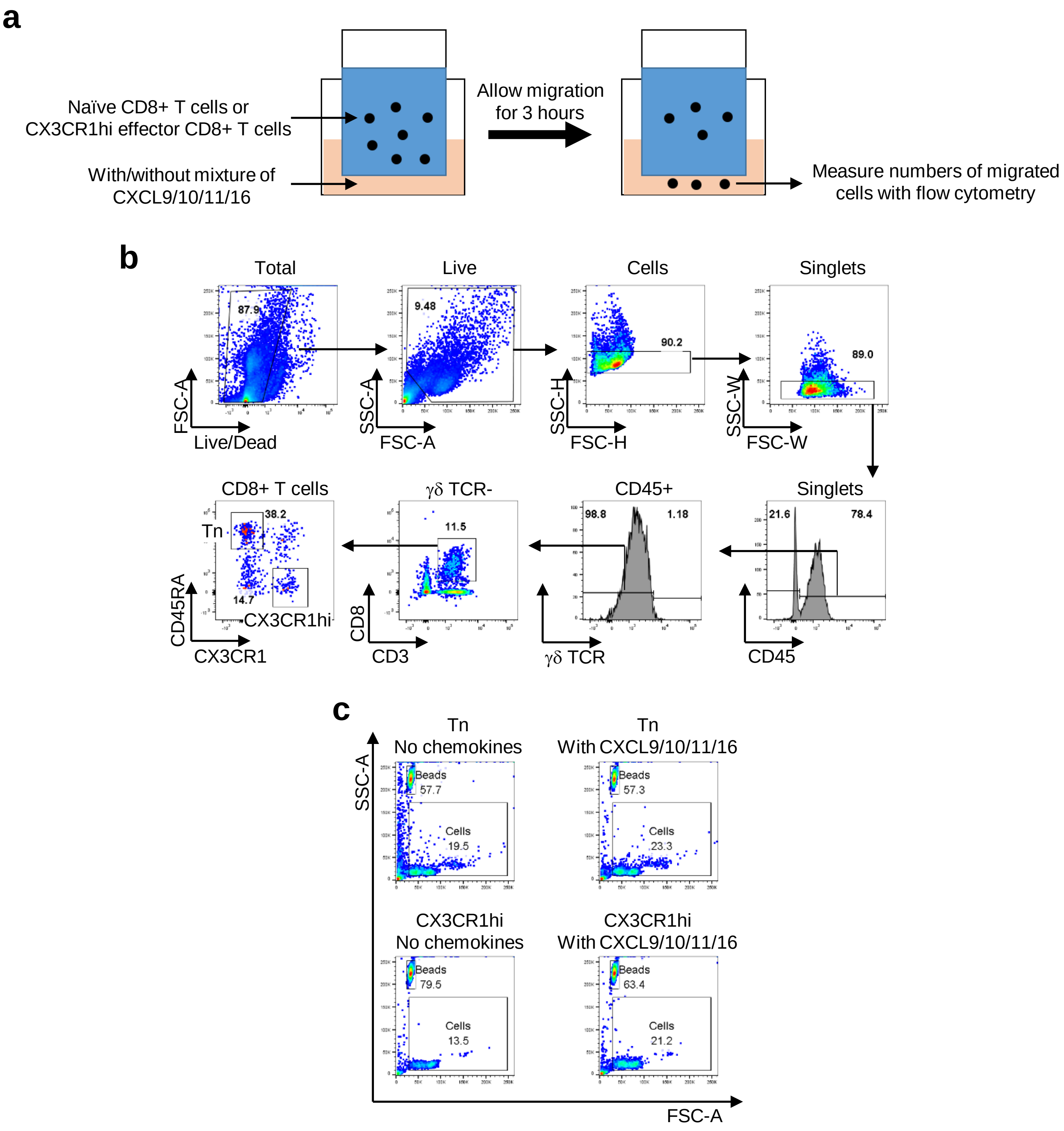

Supplementary Figure 5. CX3CR1hi effector CD8+ T cells migrated in response to CXCL9/10/11/16. (**a**) Schematics showing the experimental designs. (**b**) Flow cytometry plots showing sorting strategies. Corresponds to Figure 4. (**c**) After allowing migration of naïve or CX3CR1hi effector CD8+ T cells for three hours in the presence/absence of CXCL9/10/11/16, the cells in the lower chamber were collected. Representative flow cytometry plots taken at the time when 5,000 magnetic beads were collected. Tn, naïve CD8+ T cells; CX3CR1hi, CX3CR1hi effector CD8+ T cells.

Supplementary Figure 6

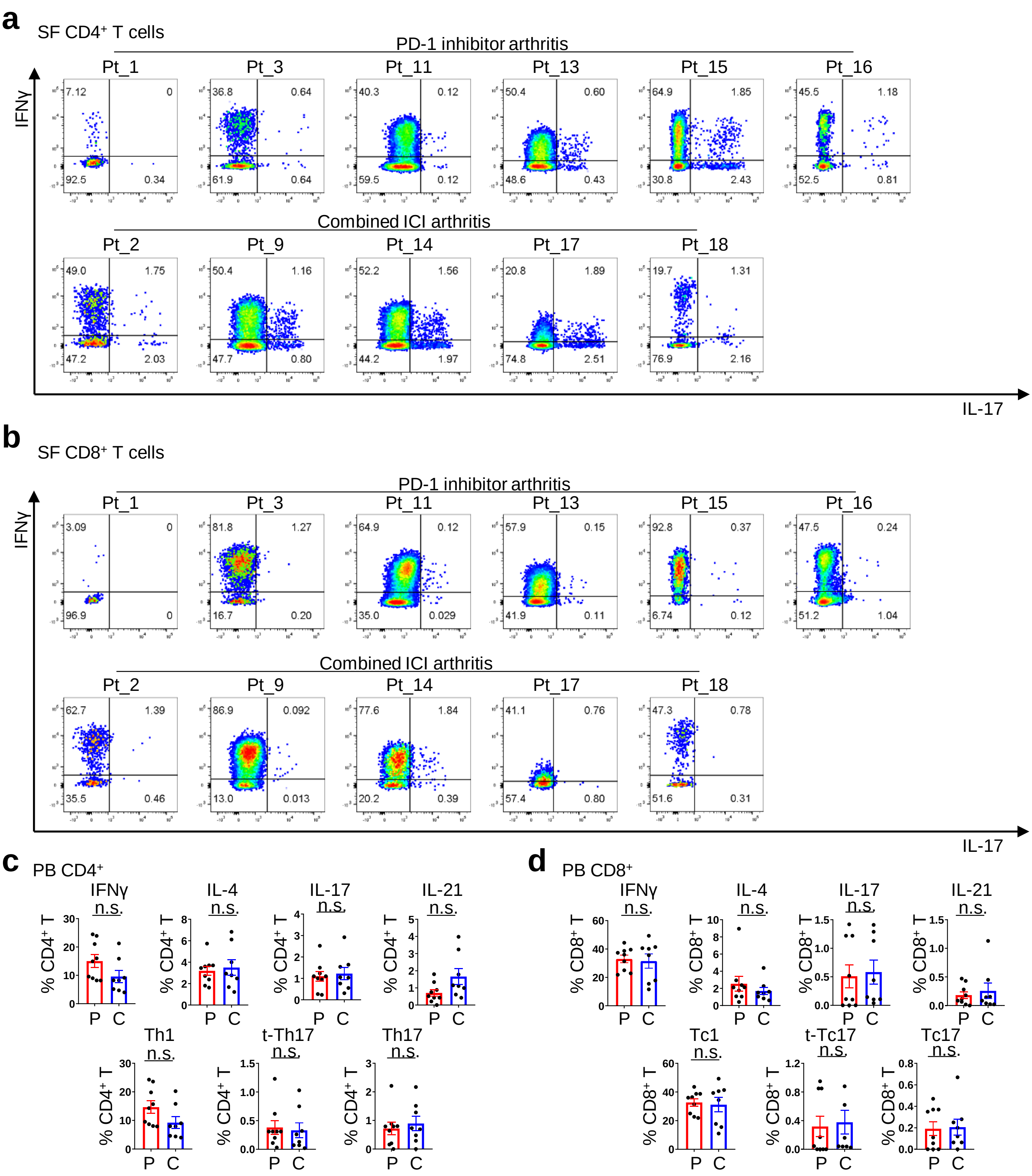

Supplementary Figure 6. Analysis of T cells producing key effector cytokines. (**a-b**) Intracellular staining of CD4+ T cells (**a**) and CD8+ T cells (**b**) in synovial fluid (SF) of individual patients who developed arthritis after PD-1 inhibitor monotherapy (PD-1 inhibitor arthritis) or combined immune checkpoint inhibitor (ICI) therapy (combined ICI arthritis). IFNγ, interferon gamma; IL, interleukin. (**c-d**) Proportion of cytokine-producing CD4+ T cells (**c**) and CD8+ T cells (**d**) in peripheral blood (PB; upper panels) and proportions of Th1/Tc1, transient (t-)Th17/t-Tc17, and Th17/Tc17 cells (lower panels). Two-sided unpaired t-test. Bars indicate the mean and SEM. P, PD-1 inhibitor arthritis; C, combined ICI arthritis. n=9 PB samples from the PD-1 inhibitor arthritis group and 8 PB samples from the combined ICI arthritis group. Source data are provided as a Source Data file.

Supplementary Figure 7

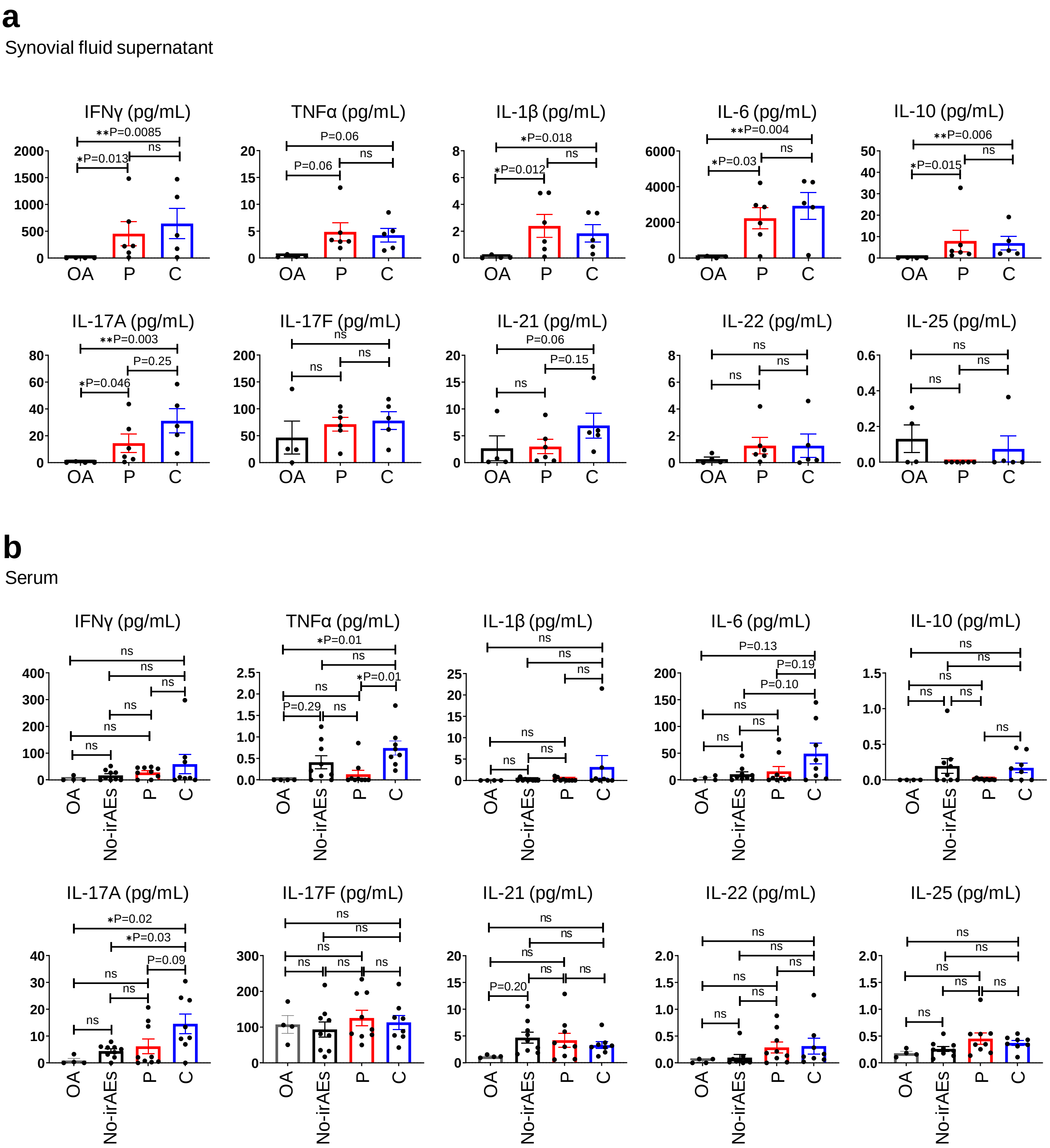

Supplementary Figure 7. Quantification of inflammatory cytokines in SF supernatant (a) and serum (b). One-way analysis of variance. *P<0.05, **P<0.01. Bars indicate the mean and SEM. No-irAEs; patients who had no irAEs for at least 12 weeks after initiating of immune checkpoint inhibitor (ICI) therapy; OA, osteoarthritis; P, PD-1 inhibitor arthritis; C, Combined ICI arthritis. IFNγ, interferon gamma; IL, interleukin. Supplementary Figure 7a: n=4 (OA), 6 (PD-1 inhibitor arthritis), and 5 (Combined ICI arthritis); Supplementary Figure 7b: n=4 (OA), 9 (no-irAE), 9 (PD-1 inhibitor arthritis), and 8 (combined ICI arthritis). Source data are provided as a Source Data file.

Supplementary Table 1. Information of peripheral blood and synovial fluid samples[a].

	No. (%)		
**Characteristic**	**Arthritis-irAE, n=20**	**Osteoarthritis, n=4**	**No irAEs, n=9**
PB	9 (45)	0 (0)	9 (100)
SF	3 (15)	4 (100)	0 (0)
Both SF and PB	8 (40)	0 (0)	0 (0)
Median time from first ICI infusion to sample donation (range)	45.8 weeks (5.5-380.5)	N/A	N/A
Median time from development of arthritis to sample donation (range)	8.5 weeks (0.5-38.5)	N/A	N/A
Intra-articular steroids <12 weeks prior to sample donation	0 (0)	0 (0)	N/A
Systemic steroids <4 weeks prior to sample donation	8 (40)	0 (0)	0 (0)
Systemic steroids at the time of sample donation	2 (10)	0 (0)	0 (0)
Characterization of SF			
Median white blood cell count (range)	4,800 cells/µL (774-46,360)	643 cells/µL (22-643)	N/A
Median percentage of neutrophils (range)	75.0% (0-89)	3% (2-14)	N/A
Median percentage of lymphocytes (range)	13.5% (4-82)	54% (32-82)	N/A
Median percentage of histiocytes (range)	10.5% (4-26)	43% (4-63)	N/A
Crystal positive	0 (0)	0 (0)	N/A
Cultures positive	0 (0)	0 (0)	N/A

[a]Abbreviations: PB, peripheral blood; SF, synovial fluid; irAE, immune-related adverse event; ICI, immune checkpoint inhibitor; N/A, not applicable.

Supplementary Table 2. Demographic and clinical characteristics of patients with PD-1 inhibitor arthritis and patients with combined ICI arthritis[a].

Characteristic	No. (%)	
	**PD-1 inhibitor arthritis, n=11**	**Combined ICI arthritis, n=9**
Median age (range)	57 years (34-73)	56 years (42-77)
Male	7 (63)	7 (78)
Median BMI (range)	28.2 (23.5-37.0)	28.8 (21.7-37.9)
Tumor type		
Melanoma	4 (36)	7 (77)
RCC	4 (36)	0 (11)
NSCLC	3 (27)	1 (11)
Neuroendocrine tumor	0 (0)	1 (11)
Pattern of arthritis		
Rheumatoid arthritis-like	3 (27)	3 (33)
Reactive arthritis-like	8 (73)	6 (67)
CTCAE grade II-IV irAEs prior to the arthritis		
Colitis	3 (27)	4 (44)
Myocarditis	1 (9)	0 (0)
Type I diabetes mellitus	1 (9)	0 (0)
Hypothyroidism	0 (0)	1 (11)
Neuritis	1 (9)	0 (0)
Median time from first ICI infusion to arthritis symptoms (range)	28.5 weeks (6-70)	87 weeks (0.5-365)
Stopped ICI therapy before arthritis development	4 (36)	3 (33)
Colitis-irAE	1 (9)	0 (0)
Myocarditis-irAE	1 (9)	0 (0)
Neuritis-irAE	1 (9)	0 (0)
Tumor progression	1 (9)	1 (11)
Completion of ICI therapy	0 (0)	2 (22)
Arthritis-irAE CTCAE grade		
II	10 (80)	6 (67)
III	1 (7)	3 (33)
Median CDAI (range)	22.0 (17-25)	20 (7-60)
Median ESR[b] (range)	44 mm/hour (8-95)	58 mm/hour (21-108)
Median CRP[c] (range)	44.7 mg/L (6.8-249)	137 mg/L (6.5-300)
ANA-positive	3 (27)	0 (0)
RF-positive	0 (0)	2 (22)
Anti-CCP antibody–positive	0 (0)	0 (0)

[a]Abbreviations: ICI, immune checkpoint inhibitor; BMI, body mass index; RCC, renal cell carcinoma; NSCLC, non-small cell lung carcinoma; CTCAE, common terminology criteria for adverse events; irAE, immune-related adverse event; CDAI, clinical disease activity index; ESR, erythrocyte sedimentation rate; CRP, C-reactive protein; ANA, anti-nuclear antibody; RF, rheumatoid factor; CCP, cyclic citrullinated peptide.
[b]Normal values of ESR range from 0 to 20.
[c]Normal values of CRP range from 0 to 10.
