# Supplementary material for: Distinct molecular and immune hallmarks of inflammatory arthritis induced by immune checkpoint inhibitors for cancer therapy
Source: Nat Commun. 2022 Apr 12;13:1970. doi: 10.1038/s41467-022-29539-3 (PMC9005525; doi:10.1038/s41467-022-29539-3)
Supplement: Supplementary file 2 — Reporting Summary [file 41467_2022_29539_MOESM2_ESM.pdf]

nature portfolio

Corresponding author(s): Linghua Wang; Roza Nurieva

Last updated by author(s): December 20, 2021

Reporting Summary

Nature Portfolio wishes to improve the reproducibility of the work that we publish. This form provides structure for consistency and transparency in reporting. For further information on Nature Portfolio policies, see our Editorial Policies and the Editorial Policy Checklist.

Statistics

For all statistical analyses, confirm that the following items are present in the figure legend, table legend, main text, or Methods section.

n/a	Confirmed	
[ ]	[x]	The exact sample size (*n*) for each experimental group/condition, given as a discrete number and unit of measurement
[ ]	[x]	A statement on whether measurements were taken from distinct samples or whether the same sample was measured repeatedly
[ ]	[x]	The statistical test(s) used AND whether they are one- or two-sided<br>*Only common tests should be described solely by name; describe more complex techniques in the Methods section.*
[x]	[ ]	A description of all covariates tested
[ ]	[x]	A description of any assumptions or corrections, such as tests of normality and adjustment for multiple comparisons
[ ]	[x]	A full description of the statistical parameters including central tendency (e.g. means) or other basic estimates (e.g. regression coefficient) AND variation (e.g. standard deviation) or associated estimates of uncertainty (e.g. confidence intervals)
[ ]	[x]	For null hypothesis testing, the test statistic (e.g. *F*, *t*, *r*) with confidence intervals, effect sizes, degrees of freedom and *P* value noted<br>*Give P values as exact values whenever suitable.*
[x]	[ ]	For Bayesian analysis, information on the choice of priors and Markov chain Monte Carlo settings
[x]	[ ]	For hierarchical and complex designs, identification of the appropriate level for tests and full reporting of outcomes
[x]	[ ]	Estimates of effect sizes (e.g. Cohen's *d*, Pearson's *r*), indicating how they were calculated

Our web collection on statistics for biologists contains articles on many of the points above.

Software and code

Policy information about availability of computer code

Data collection	BD FACs DIVA (version 8.0.1; http://www.bdbiosciences.com)
Data analysis	- Flowjo (V10.6 Tree Star http://www.flowjo.com) was used to analyze flow cytometry data.<br>- GraphPad Prism 8 (http://www.graphpad.com/scientific-software/prism/) was used to visualize flow cytometry and multiplex data.<br>- Raw single-cell RNA-seq data were processed using Cell Ranger (v3.1.0; http://www.support.10xgenomics.com/single-cell-gene-expression/software/downloads/latest), including demultiplexing the FASTQ reads, aligning them to the human reference genome (GRCh38, v3.0.0, from 10X Genomics), and counting the unique molecular identifier (UMI).<br>- In Analyzing scRNAsequencing data, Scrublet (Github last commit [https://github.com/AllonKleinLab/scrublet]), an algorithm to predict doublets in scRNA-seq data, was applied to further identify and clean doublets. We also used “RunHarmony” function of “harmony” R package (v1.0 [https://github.com/immunogenomics/harmony]) to offset the batch effect.<br>- For visualization of scRNAsequencing data, Seurat (v3.2.2; https://github.com/satijalab/seurat) was applied to the normalized gene-cell matrix to identify highly variable genes (HVGs) for unsupervised cell clustering. The dimensionality was further reduced using Uniform Manifold Approximation and Projection (UMAP; https://github.com/lmcinnes/umap) method.<br>- For chemokine and chemokine receptor analyses out of scRNAsequencing data, iTALK (http://github.com/Coolgenome/iTALK) was used.<br>- Raw T cell receptor (TCR) sequencing data were processed by using 10X Genomics Cell Ranger (v3.1.0).

For manuscripts utilizing custom algorithms or software that are central to the research but not yet described in published literature, software must be made available to editors and reviewers. We strongly encourage code deposition in a community repository (e.g. GitHub). See the Nature Portfolio guidelines for submitting code & software for further information.

Data

Policy information about availability of data

All manuscripts must include a data availability statement. This statement should provide the following information, where applicable:

- Accession codes, unique identifiers, or web links for publicly available datasets
- A description of any restrictions on data availability
- For clinical datasets or third party data, please ensure that the statement adheres to our policy

The scRNAseq data and TCRseq data generated in this study have been deposited in the GEO database under accession code GSE173303 (https://www.ncbi.nlm.nih.gov/geo/query/acc.cgi?acc=GSE173303). The human reference genome data (GRCh38, v3.0.0, from 10X Genomics) are available at https://www.ncbi.nlm.nih.gov/assembly/GCF_000001405.26/. The remaining data are available within the article, Supplementary Information or Source Data file.

Field-specific reporting

Please select the one below that is the best fit for your research. If you are not sure, read the appropriate sections before making your selection.

[x] Life sciences [ ] Behavioural & social sciences [ ] Ecological, evolutionary & environmental sciences

For a reference copy of the document with all sections, see nature.com/documents/nr-reporting-summary-flat.pdf

Life sciences study design

All studies must disclose on these points even when the disclosure is negative.

Sample size	Sample sizes were determined with the reference, which performed scRNAsequencing of blood and matching synovial fluid and shown to be of sufficient size of sample to discriminate major immune cell subsets (Penkava et al. PMID: 32958743).
Data exclusions	We carried out a number of steps to filter out poor quality data. First, we removed cells with high mitochondrial gene expression because damaged and dead cells often exhibit extensive mitochondrial contamination. Specifically, we calculate the proportion of UMIs from mitochondrial genes (all genes with names start with “MT-” or “mt-”) for each cell, then remove cells which contain more than 15%mitochondrial UMIs. Second, we removed cells for which less than 200 genes were detected. Third, doublets were identified using a multi-step approach: 1) library complexity: cells with high complexity libraries (in which detected transcripts are aligned to more than 6500 genes) were removed; 2) Cluster distribution: doublets or multiplets likely form distinct clusters with hybrid expression features and exhibit an aberrantly high gene count; 3) cluster marker gene expression: cells of a cluster express markers from distinct lineages (e.g., cells in the T-cell cluster showed expression of myeloid cell markers and vice versa); 4) doublet detection algorithm: scrublet, an algorithm to predict doublets in scRNA-seq data, was applied to further identify and clean doublets that could have been missed by steps 1-3.
Replication	Single cell RNA sequencing from synovial fluid and matching peripheral blood was performed three times for 10X sequencing. Cell numbers as well as data at each time were comparable. To validate the data of single cell RNA sequencing, we performed flow cytometry.<br>Flow cytometry (n=10), multiplex (n=3), HLA B27 typing (n=2), Treg suppression assay (n=4), and migration assay (n=2) were performed multiple times independantly as indicated by n. All replication attempts were successful.
Randomization	Because this was a prospective observational study, randomization was not necessary.
Blinding	Because this was a prospective observational study, blinding was not necessary.

Reporting for specific materials, systems and methods

We require information from authors about some types of materials, experimental systems and methods used in many studies. Here, indicate whether each material, system or method listed is relevant to your study. If you are not sure if a list item applies to your research, read the appropriate section before selecting a response.

Materials & experimental systems

n/a	Involved in the study	
[ ]	[x]	Antibodies
[x]	[ ]	Eukaryotic cell lines
[x]	[ ]	Palaeontology and archaeology
[x]	[ ]	Animals and other organisms
[ ]	[x]	Human research participants
[x]	[ ]	Clinical data
[x]	[ ]	Dual use research of concern

Methods

n/a	Involved in the study	
[x]	[ ]	ChIP-seq
[ ]	[x]	Flow cytometry
[x]	[ ]	MRI-based neuroimaging

Antibodies

Antibodies used	Anti-Human CD16 BUV 395 BD Biosciences Cat#: 563785 RRID: AB_2744293, Dilution 1:20<br>Anti-Human CD27 BUV 395 BD Biosciences Cat#: 563815 RRID: AB_2744349, Dilution 1:50<br>Anti-Human CD4 BUV 395 BD Biosciences Cat#: 563550 RRID: AB_2738273, Dilution 1:50

Anti-Human CD56 Brilliant Violet 421 Biolegend Cat#: 362552 RRID: AB_2566061, Dilution 1:50
Anti-Human gd TCR Brilliant Violet 421 Biolegend Cat#: 331218 RRID: AB_2562317, Dilution 1:50
Anti-Human IL-4 Brilliant Violet 421 Biolegend Cat#: 500826 RRID: AB_2561679, Dilution 1:50
Anti-Human CCR7 Brilliant Violet 421 Biolegend Cat#: 353208 RRID: AB_11203894, Dilution 1:50
Anti-Human Ki67 Brilliant Violet 421 Biolegend Cat#: 652411 RRID: AB_2562663, Dilution 1:50
Anti-Human CD19 Brilliant Violet 785 Biolegend Cat#: 302240 RRID: AB_2563442, Dilution 1:50
Anti-Human CD45RA Brilliant Violet 785 Biolegend Cat#: 304140 RRID: AB_2563816, Dilution 1:50
Anti-Human PD-1 Brilliant Violet 785 Biolegend Cat#: 367432 RRID: AB_2721562, Dilution 1:50
Anti-Human CD3 PerCP Biolegend Cat#: 344808 RRID: AB_10640736, Dilution 1:50
Anti-Human HLA-DR FITC Biolegend Cat#: 307620 RRID: AB_493175, Dilution 1:50
Anti-Human CD56 FITC Biolegend Cat#: 318304 RRID: AB_604100, Dilution 1:50
Anti-Human FoxP3 FITC Biolegend Cat#: 320106 RRID: AB_439752, Dilution 1:25
Anti-Human CD123 PE Biolegend Cat#: 306006 RRID: AB_314580, Dilution 1:50
Anti-Human CD19 PE Biolegend Cat#: 302208 RRID: AB_314238, Dilution 1:50
Anti-Human IL-21 PE BD Biosciences Cat#: 562042 RRID: AB_10896123, Dilution 1:20
Anti-Human CD25 PE Biolegend Cat#: 356104 RRID: AB_2561861, Dilution 1:50
Anti-Human CX3CR1 PE Biolegend Cat#: 355704 RRID: AB_2561681, Dilution 1:50
Anti-Human CD24 PE-Dazzle Biolegend Cat#: 311134 RRID: AB_2566349, Dilution 1:50
Anti-Human CTLA-4 PE-Dazzle Biolegend Cat#: 369616 RRID: AB_2632878, Dilution 1:25
Anti-Human IFNg PE-Dazzle Biolegend Cat#:502546 RRID: AB_2563627, Dilution 1:25
Anti-Human CD11c PE-Cy7 Biolegend Cat#: 337216 RRID: AB_2129790, Dilution 1:20
Anti-Human CCR7 PE-Cy7 Biolegend Cat#: 353226 RRID: AB_11126145, Dilution 1:50
Anti-Human CD25 PE-Cy7 Biolegend Cat#: 302612 RRID: AB_314282, Dilution 1:50
Anti-Human IL-17A PE-Cy7 Biolegend Cat#: 512315 RRID: AB_2295923, Dilution 1:40
Anti-Human CD4 PE-Cy7 Biolegend Cat#: 30051 RRID: AB_314080, Dilution 1:50
Anti-Human CD4 APC Biolegend Cat#: 300514 RRID: AB_314082, Dilution 1:50
Anti-Human IL-10 APC Biolegend Cat#: 506806 RRID: AB_315456, Dilution 1:40
Anti-Human IL-2 APC Biolegend Cat#: 500310 RRID: AB_315097, Dilution 1:25
Anti-Human CD14 Alexa Fluor 700 Biolegend Cat#: 301822 RRID: AB_493747, Dilution 1:50
Anti-Human CD8 Alexa Fluor 700 Biolegend Cat#: 300920 RRID: AB_528885, Dilution 1:50
Anti-Human CD127 Alexa Fluor 700 Biolegend Cat#: 351344 RRID: AB_2566200, Dilution 1:50
Anti-Human CD45 APC-Fire Biolegend Cat#: 368518 RRID: AB_2616705, Dilution 1:50

Validation

All antibodies were validated as described on the websites of Biolegend and BD Biosciences.

Anti-Human CD16 BUV 395 BD Biosciences Cat#: 563785
https://www.bdbiosciences.com/en-us/search-results?searchKey=563785

Anti-Human CD27 BUV 395 BD Biosciences Cat#: 563815
https://www.bdbiosciences.com/en-us/search-results?searchKey=563815

Anti-Human CD4 BUV 395 BD Biosciences Cat#: 563550
https://www.bdbiosciences.com/en-us/search-results?searchKey=563550

Anti-Human CD56 Brilliant Violet 421 Biolegend Cat#: 362552
https://www.biolegend.com/en-us/products/brilliant-violet-421-anti-human-cd56-ncam-antibody-12179

Anti-Human gd TCR Brilliant Violet 421 Biolegend Cat#: 331218
https://www.biolegend.com/en-us/products/brilliant-violet-421-anti-human-tcr-gamma-delta-antibody-8705

Anti-Human IL-4 Brilliant Violet 421 Biolegend Cat#: 500826
https://www.biolegend.com/en-us/products/brilliant-violet-421-anti-human-il-4-antibody-7224

Anti-Human CCR7 Brilliant Violet 421 Biolegend Cat#: 353208
https://www.biolegend.com/en-us/products/brilliant-violet-421-anti-human-cd197-ccr7-antibody-7497

Anti-Human Ki67 Brilliant Violet 421 Biolegend Cat#: 652411
https://www.biolegend.com/en-us/products/brilliant-violet-421-anti-mouse-ki-67-antibody-8982

Anti-Human CD19 Brilliant Violet 785 Biolegend Cat#: 302240
https://www.biolegend.com/en-us/products/brilliant-violet-785-anti-human-cd19-antibody-7967

Anti-Human CD45RA Brilliant Violet 785 Biolegend Cat#: 304140
https://www.biolegend.com/en-us/products/brilliant-violet-785-anti-human-cd45ra-antibody-7972

Anti-Human PD-1 Brilliant Violet 785 Biolegend Cat#: 367432
https://www.biolegend.com/en-us/products/brilliant-violet-785-anti-human-cd279-pd-1-antibody-15317

Anti-Human CD3 PerCP Biolegend Cat#: 344808
https://www.biolegend.com/en-us/products/percp-cyanine5-5-anti-human-cd3-antibody-6932

Anti-Human HLA-DR FITC Biolegend Cat#: 307620
https://www.biolegend.com/en-us/products/alexa-fluor-488-anti-human-hla-dr-antibody-3146

Anti-Human CD56 FITC Biolegend Cat#: 318304
https://www.biolegend.com/en-us/products/fitc-anti-human-cd56-ncam-antibody-3795

Anti-Human FoxP3 FITC Biolegend Cat#: 320106
https://www.biolegend.com/en-us/products/fitc-anti-human-foxp3-antibody-2946

Anti-Human CD123 PE Biolegend Cat#: 306006
https://www.biolegend.com/en-us/products/pe-anti-human-cd123-antibody-577

Anti-Human CD19 PE Biolegend Cat#: 302208
https://www.biolegend.com/en-us/products/pe-anti-human-cd19-antibody-719

Anti-Human IL-21 PE BD Biosciences Cat#: 562042
https://www.bdbiosciences.com/en-us/search-results?searchKey=562042

Anti-Human CD25 PE Biolegend Cat#: 356104
https://www.biolegend.com/en-us/products/pe-anti-human-cd25-antibody-8389

Anti-Human CX3CR1 PE Biolegend Cat#: 355704
https://www.biolegend.com/en-us/products/pe-anti-human-cx3cr1-antibody-8280

Anti-Human CD24 PE-Dazzle Biolegend Cat#: 311134
https://www.biolegend.com/en-us/products/pe-dazzle-594-anti-human-cd24-antibody-12388

Anti-Human CTLA-4 PE-Dazzle Biolegend Cat#: 369616
https://www.biolegend.com/en-us/products/pedazzle-594-anti-human-cd152-ctla-4-antibody-13817

Anti-Human IFNg PE-Dazzle Biolegend Cat#:502546
https://www.biolegend.com/en-us/products/pe-dazzle-594-anti-human-ifn-gamma-antibody-9786

Anti-Human CD11c PE-Cy7 Biolegend Cat#: 337216
https://www.biolegend.com/en-us/products/pe-cyanine7-anti-human-cd11c-antibody-6129

Anti-Human CCR7 PE-Cy7 Biolegend Cat#: 353226
https://www.biolegend.com/en-us/products/pecyanine7-anti-human-cd197-ccr7-antibody-7694

Anti-Human CD25 PE-Cy7 Biolegend Cat#: 302612
https://www.biolegend.com/en-us/products/pe-cyanine7-anti-human-cd25-antibody-1909

Anti-Human IL-17A PE-Cy7 Biolegend Cat#: 512315
https://www.biolegend.com/en-us/products/pe-cyanine7-anti-human-il-17a-antibody-5954

Anti-Human CD4 PE-Cy7 Biolegend Cat#: 300512
https://www.biolegend.com/en-us/products/pe-cyanine7-anti-human-cd4-antibody-829

Anti-Human CD4 APC Biolegend Cat#: 300514
https://www.biolegend.com/en-us/products/apc-anti-human-cd4-antibody-823

Anti-Human IL-10 APC Biolegend Cat#: 506806
https://www.biolegend.com/en-us/products/apc-anti-human-il-10-antibody-1572

Anti-Human IL-2 APC Biolegend Cat#: 500310
https://www.biolegend.com/en-us/products/apc-anti-human-il-2-antibody-1348

Anti-Human CD14 Alexa Fluor 700 Biolegend Cat#: 301822
https://www.biolegend.com/en-us/products/alexa-fluor-700-anti-human-cd14-antibody-3397

Anti-Human CD8 Alexa Fluor 700 Biolegend Cat#: 300920
https://www.biolegend.com/en-us/products/alexa-fluor-700-anti-human-cd8a-antibody-3434

Anti-Human CD127 Alexa Fluor 700 Biolegend Cat#: 351344
https://www.biolegend.com/en-us/products/alexa-fluor-700-anti-human-cd127-il-7ralpha-antibody-12275

Anti-Human CD45 APC-Fire Biolegend Cat#: 368518
https://www.biolegend.com/en-us/products/apc-fire-750-anti-human-cd45-antibody-13178

Human research participants

Policy information about studies involving human research participants

Population characteristics	We collected residual synovial fluid (SF) and/or peripheral blood (PB) from 20 patients who newly developed arthritis after immune checkpoint inhibitor (ICI) therapy. As serum-negative controls, we collected PB samples from patients who had not developed irAEs at least 12 weeks after initiating ICI therapy. For SF supernatant-negative controls, we collected SF samples from patients with osteoarthritis. The patients met the American College of Rheumatology diagnostic criteria for osteoarthritis. Participants included both male and female patients ranging in age from 34 to 77 years. Detailed information about individual participants can be found in Table 1 and Supplementary Data file 1.
Recruitment	The patients were recruited prospectively from Rheumatology services (either outpatient or inpatient settings) at the University of Texas MD Anderson Cancer Center. The diagnosis of inflammatory arthritis was determined by a history and physical exam performed by a treating rheumatologist at MD Anderson (S.T.K., M.S.-A, J.H.T., and H.L.). Prior to the procedures (diagnostic arthrocentesis and/or venipuncture), participants provided written informed consent, allowing collection of residual SF and/or PB samples as well as prospective follow-up for 12 months after the sample donation. Because mild arthritis-irAE is likely managed by either a patient or by an oncologist, recruitment in this study may be biased to more severe arthritis-irAE and the results might have revealed an altered immunity of severe inflammation.
Ethics oversight	The study was approved by the institutional review board at The University of Texas MD Anderson Cancer Center (IRB No: PA16-0935).

Note that full information on the approval of the study protocol must also be provided in the manuscript.

Clinical data

Policy information about clinical studies
All manuscripts should comply with the ICMJEguidelines for publication of clinical research and a completedCONSORT checklist must be included with all submissions.

Clinical trial registration	This it not a clinical trial.
Study protocol	After residual synovial fluid and/or peripheral blood samples were donated, the participants were prospectively followed up for 12 months.
Data collection	Information on age, sex, body mass index, tumors, pattern of arthritis, history of irAEs prior to the arthritis, onset of the arthritis, CDAI, erythrocyte sedimentation rate, C-reactive protein, anti-nuclear antibody, rheumatoid factor, and anti-cyclic citrullinated peptide antibody were obtained from the medical record. We followed patients with arthritis-irAE for 12 months after the sample collection whether the patients failed steroid monotherapy and required steroid-sparing disease modifying anti-rheumatic drugs (DMARDs).

Outcomes	At the end of the follow-up (12 months after the sample donation), we calculated percentages of patients who required DMARDs in PD-1 inhibitor arthritis and combined PD-1 and CTLA-4 inhibitor arthritis groups.

Flow Cytometry

Plots

Confirm that:

- [x] The axis labels state the marker and fluorochrome used (e.g. CD4-FITC).
- [x] The axis scales are clearly visible. Include numbers along axes only for bottom left plot of group (a 'group' is an analysis of identical markers).
- [x] All plots are contour plots with outliers or pseudocolor plots.
- [x] A numerical value for number of cells or percentage (with statistics) is provided.

Methodology

Sample preparation	Cryopreserved SF cells and PBMCs were thawed, washed, and stained with flow cytometry antibodies to CD3, CD4, CD8, CD11b, CD11c, CD14, CD16, CD19, CD25, CD45, CD45A, CD56, CD127, CCR7, HLA-DR, PD-1, CTLA-4, and γδ TCR. For cytokine intracellular staining, SF cells and PBMCs were stimulated with cell activation cocktail (Biolegend) containing phorbol 12-myristate 13-acetate, ionomycin, and brefeldin for 4 hours. Cells were stained for surface molecules, fixed with BD Fixation/ Permeabilization solution, permeabilized with BD PERM/WashTM buffer, and stained with antibodies to IFNγ, IL-4, IL-10, IL-17A, and IL-21 For FoxP3 and Ki67 staining, cells were stained for surface molecules, fixed, and permeabilized with eBioscienceTM FoxP3/Transcription staining buffer set. Subsequently, the cells were stained for FoxP3 and Ki67. Stained samples were acquired using LSR II FORTESSA X-20 (BD Biosciences) and analyzed with FlowJo software (TreeStar).
Instrument	LSR II FORTESSA X-20 (BD Biosciencees)
Software	Flowjo software (TreeStar)
Cell population abundance	Lymphocytes: ~60% of all events; single cells ~ 96.6% of lymphocytes.
Gating strategy	Live immune cells were detected by gating CD45 and live-dead. Single cells were gated with forward and side scatters. Using well-known lineage markers, we identified major immune cells including macrophages, neutrophils, myeloid dendritic cells, plasmacytoid dendritic cells, natural killer cells, B cells, T cells, and gamma delta T cells. The flow plots showing gate strategy is available in Extended Data Figure 1.

- [x] Tick this box to confirm that a figure exemplifying the gating strategy is provided in the Supplementary Information.
