# Supplementary material for: Distinct molecular and immune hallmarks of inflammatory arthritis induced by immune checkpoint inhibitors for cancer therapy
Source: Nat Commun. 2022 Apr 12;13:1970. doi: 10.1038/s41467-022-29539-3 (PMC9005525; doi:10.1038/s41467-022-29539-3)
Supplement: Supplementary file 4 — Description of Additional Supplementary Files [file 41467_2022_29539_MOESM4_ESM.pdf]

Title: Supplementary Data file 1.
Description: Demographic and clinical characteristics, including outcome of arthritis as an immune-related adverse event, of individual patients in the study.

Title: Supplementary Data file 2.
Description: Differentially expressed genes in live cell clusters. Pvalue was calculated using Wilcoxon Rank Sum test. P-adj value is false discovery rate adjusted P-value.

Title: Supplementary Data file 3.
Description: Differentially expressed genes in natural killer cell, natural killer T cell, and T cell subclusters. P-value was calculated using Wilcoxon Rank Sum test. P-adj value is false discovery rate adjusted P-value.

Title: Supplementary Data file 4.
Description: Differentially expressed genes in regulatory T cell subclusters. P-value was calculated using Wilcoxon Rank Sum test. P-adj value is false discovery rate adjusted P-value.

Title: Supplementary Data file 5.
Description: Differentially expressed genes in regulatory T cell. Pvalue was calculated using Wilcoxon Rank Sum test. P-adj value is false discovery rate adjusted P-value.
